# Effect of cocoa (*Theobroma cacao* L.) on platelet function testing profiles in patients with coronary artery disease: ECLAIR pilot study

**DOI:** 10.1136/openhrt-2022-002066

**Published:** 2022-09-13

**Authors:** Naveen Anand Seecheran, Darin Sukha, Kathryn Grimaldos, Gabriella Grimaldos, Srivane Richard, Aleena Ishmael, Ceylon Gomes, Lirmala Kampradi, Rajeev Seecheran, Valmiki Seecheran, Lakshmipathi Peram, Darren Dookeeram, Stanley Giddings, Sherry Sandy, Anil Ramlackhansingh, Sadi Raza, Pathmanathan Umaharan, Antonio Tello-Montoliu, David Schneider

**Affiliations:** 1 Clinical Medical Sciences, The University of the West Indies, Saint Augustine, Trinidad and Tobago; 2 Cocoa Research Centre, The University of the West Indies, Saint Augustine, Trinidad and Tobago; 3 Department of Medicine, North Central Regional Health Authority, Champ Fleurs, Trinidad and Tobago; 4 Department of Medicine, University of Kansas Medical Center, Wichita, Kansas, USA; 5 Cardiology Division, HeartPlace, Dallas, Texas, USA; 6 Cardiology Division, Universidad de Murcia, Murcia, Spain; 7 Cardiology Division, University of Vermont Medical Center, Burlington, Vermont, USA

**Keywords:** Epidemiology, Pharmacology, Clinical, Drug Interactions

## Abstract

**Introduction:**

This prospective pharmacodynamic nutraceutical study assessed the effect of a 1-week trial of 30 g/day of 65% cocoa (dark chocolate) (*Theobroma cacao* L.) consumption intervention on platelet reactivity.

**Methods:**

Patients with stable coronary artery disease (CAD) (n=20) who were on maintenance dual antiplatelet therapy of aspirin (ASA) 81 mg/day and clopidogrel 75 mg/day were recruited. Platelet function was evaluated with the VerifyNow P2Y_12_ reaction unit (PRU) and aspirin reaction unit (ARU) assays (Werfen, Bedford, Massachusetts, USA) and assessed prior to initiation of and after a 1-week trial of 30 g/day of 65% cocoa consumption intervention. Results were compared with a paired t-test.

**Results:**

Cocoa augmented the inhibitory effect of clopidogrel, demonstrated by a reduction of 11.9% (95% CI 5.7% to 18.0%, p value 0.001), significantly decreasing the PRU by 26.85 (95% CI 12.22 to 41.48, p value 0.001). The inhibitory effect of ASA was not impacted by cocoa, reflected by a non-significant reduction in ARU of 17.65 (95% CI 21.00 to 56.3, p value 0.351). No patients experienced any serious adverse events.

**Conclusions:**

Cocoa augmented the inhibitory effect of clopidogrel but not ASA. This nutraceutical study could be potentially informative and applicable for patients with stable CAD. Further long-term studies are required to confirm these exploratory findings.

**Trial registration number:**

NCT04554901.

**WHAT IS ALREADY KNOWN ON THIS TOPIC**

- Cardiovascular (CV) diseases, principally coronary artery disease (CAD) and cerebrovascular events, are the leading cause of global mortality and a major contributor to disability.
- Cocoa (*Theobroma cacao* L.) is a rich source of bioactive compounds such as flavonoids, and its consumption has been associated with favourable nutraceutical effects, such as the positive modulation of platelet-mediated haemostasis.
- Several observational studies have demonstrated attenuated CV mortality with cocoa consumption; however, these nutraceutical effects are still controversial as cocoa may conversely accentuate CV risk due to adverse glycaemic and lipidaemic effects.

**WHAT THIS STUDY ADDS**

- Cocoa augmented the inhibitory effect of clopidogrel, demonstrated by a reduction of 11.9% (95% CI 5.7% to 18.0%, p value 0.001), significantly decreasing the PRU by 26.85 (95% CI 12.22 to 41.48, p value 0.001). The inhibitory effect of aspirin (ASA) was not impacted by cocoa, reflected by a non-significant reduction in ASA reaction unit of 17.65 (95% CI 21.00 to 56.3, p value 0.351).

**HOW THIS STUDY MIGHT AFFECT RESEARCH, PRACTICE OR POLICY**

- This nutraceutical study could be potentially informative and applicable for patients with stable CAD on dual antiplatelet therapy with ASA and clopidogrel.

## Introduction

Cardiovascular diseases (CVDs), principally coronary artery disease (CAD) and cerebrovascular events (CVEs), are the leading cause of global mortality and a major contributor to disability.^1 2^ CVD prevalence is only likely to substantially increase due to an ageing population in low-income regions such as the Caribbean, where the share of older persons is projected to double between 2019 and 2050.^1^ Within the last decade, it has since emerged that adult mortality was chiefly attributed to the vascular disease spectrum in Trinidad and Tobago.^3 4^

Cocoa (*Theobroma cacao* L.) is a rich source of bioactive compounds, such as flavonoids, and its consumption has been associated with several favourable effects, such as the positive modulation of platelet-mediated haemostasis.^5–7^ High platelet reactivity (HPR) in patients with CAD while on chronic dual antiplatelet therapy (DAPT) is associated with a higher risk of major adverse cardiovascular events (MACE), alluding to the need for tailored antithrombotic therapies.^8 9^

Studies have demonstrated attenuated cardiovascular (CV) mortality with cocoa consumption; however, these nutraceutical effects are still controversial as cocoa may conversely accentuate CV risk due to adverse glycaemic and lipidaemic effects.^10 11^

This prospective study is novel in determining the effect of a 1-week trial of 30 g/day of 65% cocoa consumption intervention on platelet reactivity using the VerifyNow (VN) system on patients with CAD on DAPT.

## Materials

### Study design and patient population

The study complied with the Declaration of Helsinki, International Conference on Harmonisation, Good Clinical Practice, and was approved by the Campus Research Ethics Committee of the University of the West Indies, St. Augustine, Trinidad.^12^ All participants provided written informed consent to participate in a prospective, open-label study that assessed the effect of a 1-week trial of 30 g/day of 65% cocoa intervention (dark chocolate) (three 10 g bars each consumed at breakfast, lunch and dinner, with a composition of 65% cocoa solids and 35% sugar). These cocoa (dark chocolate) bars were produced by the Cocoa Research Centre at the University of the West Indies, St. Augustine, Trinidad, using single-estate origin cocoa beans sourced from Ortinola Estates, St. Joseph, Trinidad, as previously described and supervised by the lead food technologist.^13^ Patients were screened and enrolled between September 2021 and December 2021 at the cardiology outpatient clinic at our institution, Trinidad Institute of Medical Technology, Trinidad and Tobago. They were considered eligible for the study if they were above 18 years of age and awaiting elective percutaneous coronary intervention (PCI) or coronary artery bypass grafting (CABG) on DAPT for at least 4 weeks with aspirin (ASA) 81 mg/day maintenance dose and clopidogrel 75 mg/day maintenance dose. Exclusion criteria for this study included an acute coronary syndrome within 6 months, active bleeding, prior haemorrhagic CVE, clinical instability after an index event, use of an oral anticoagulation agent (warfarin derivative or other anticoagulant therapy such as dabigatran, rivaroxaban, apixaban and edoxaban), platelet count of <100×10^9^/µL, haemoglobin of <10 g/dL, serum creatinine of >1.5 mg/dL, patients on concurrent CYP 2C19 inhibitors and CYP 3A4 inducers. After completing the study, they were followed up for 28 days post procedure to assess whether they experienced any serious adverse events (SAEs).

### Blood sampling and VN aspirin reaction unit (ARU) and P2Y_12_ testing

**Figure 1:**
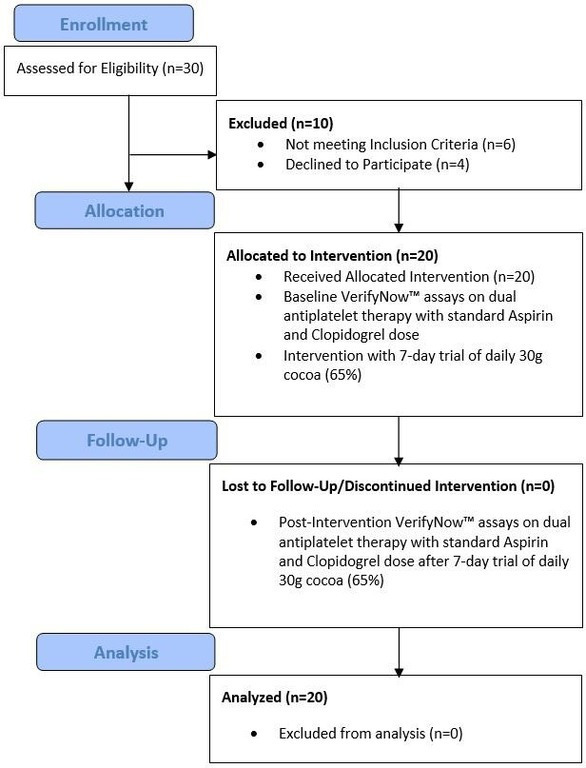
Methodology outline.

Clopidogrel was not administered on the morning of their fasting scheduled visit (08:00–09:00) (18–24 hours before baseline blood sampling), which ensured the determination of clopidogrel-induced platelet reactivity (trough). Blood samples were obtained at rest by antecubital puncture using a 21-gauge needle and placed into VACUETTE (Greiner Bio-One North America, Monroe, North Carolina, USA) blood collecting tubes containing 3.8% trisodium citrate (#454322) after discarding the first 5 mL of blood to avoid artifactual platelet activation. Samples were processed by laboratory personnel blinded to ongoing study data. The platelet function assays used were the VN P2Y_12_ reactions units (PRU) assay and ARU assay (Werfen, Bedford, Massachusetts, USA). The assays were performed according to standard protocols, as previously described.^14 15^ A PRU of >208 was considered high on-treatment platelet reactivity according to the last consensus.^16^ An ARU of ≥550 was considered ASA resistance during treatment with 81 mg.^17^ The enrolled patients were then treated with a 1-week trial of 30 g/day of 65% cocoa intervention (three 10 g bars each consumed at breakfast, lunch and dinner, with a composition of 65% cocoa solids and 35% sugar) with cocoa (dark chocolate) bar accountability by the clinical research associate. After 7 days of the cocoa intervention, platelet reactivity was assessed with both assays using the aforementioned methodology (figure 1).

### Patient interview and case report form (CRF)

The patients’ demographic data were recorded on a CRF and included the patient’s medical, procedural history and any CV medications.

### Statistical analysis

The sample size was calculated as 20 patients based on a paired proportion sample, an alpha (α) value of 0.05, power of 80%, estimated baseline prevalence of 40% of PRU of >208 and absolute delta of 20% (expected prevalence of 20% of PRU of >208). Continuous variables were expressed as means±95% CIs and categorical variables as frequencies and percentages. Paired t-tests were used to compare mean differences in PRU scores and McNemar’s test for paired proportions. No adjustments for multiple comparisons were made. Data collection was complete. A two-tailed p value of 0.05 was considered to indicate a statistically significant difference for all the analyses performed. Statistical analysis was performed using SPSS V.28.0 software.

## Results

**Figure 2:**
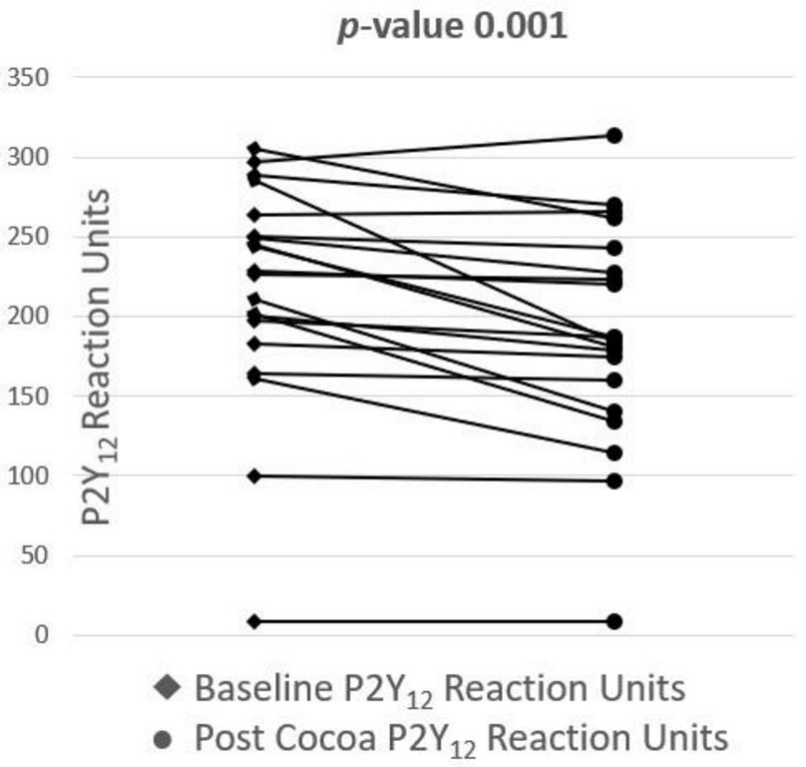
Comparison of patients’ P2Y_12_ reaction units before and after the cocoa (dark chocolate) intervention.

A total of 20 patients with stable CAD on DAPT with ASA and clopidogrel were enrolled in the study. Table 1 shows the demographics of the study participants. The mean age was 61.4 years. Of the patients, 35% were women, and 80% were South Asian in ethnicity. The mean body mass index was 28 kg/m^2^. The prevalence of prior myocardial infarction (MI) and diabetes mellitus was 50%. Twenty-five per cent PCI, with 15% prior CABG. One-quarter were on insulin therapy, while 15%, 5% and an additional 5% were on metformin, sulfonylureas and sodium–glucose cotransporter-2 inhibitors, respectively. Cocoa augmented the inhibitory effect of clopidogrel, demonstrated by a reduction of 11.9% (95% CI 5.7% to 18.0%, p value 0.001), significantly decreasing the PRU by 26.85 (95% CI 12.22 to 41.48, p value 0.001) (table 2 and figure 2). The inhibitory effect of ASA was not impacted by cocoa, reflected by a non-significant reduction in ARU of 17.65 (95% CI 21.00 to 56.3, p value 0.351; table 2). Of the 20 patients, 12 (60%) had a baseline PRU of >208 compared with 8 (40%) patients post cocoa intervention, which was non-significant (p value 0.125). Of the 20 patients, 5 (25%) had a baseline ARU of >550 which remained unchanged post cocoa intervention (non-significant). No patients experienced any SAEs.

**Table 1:** Patient population

Characteristics	Frequency (%)
Age (years)	61.4 (mean)
Gender, n (%)	
Female	7 (35)
Male	13 (65)
Ethnicity, n (%)	
South Asian	16 (80)
Caribbean black	3 (15)
Inter-racial	1 (5)
Body mass index (kg/m^2^)	28.0 (mean) (normal 18.5–24.9)
Weight (kg)	73.1
Systolic blood pressure (mm Hg)	144 (normal <120)
Diastolic blood pressure (mm Hg)	81 (normal <80)
Comorbidities, n (%)	
Prior myocardial infarction	10 (50)
Diabetes mellitus	10 (50)
Glycosylated haemoglobin (%)	8.3 (mean) (normal <6)
Fasting blood glucose (mg/dL)	182 (normal <126)
Hypertension	13 (65)
Dyslipidaemia	16 (80)
Chronic kidney disease	0 (0)
Cerebrovascular events	1 (5)
Chronic obstructive pulmonary disease	0 (0)
Peripheral artery disease	0 (0)
Cardiovascular medications, n (%)	
Aspirin	20 (100)
Clopidogrel	20 (100)
ACE inhibitor, angiotensin receptor blocker, neprilysin inhibitor	13 (65)
Beta blocker	13 (65)
Statin	18 (90)
Mineralocorticoid receptor antagonist	3 (15)
Calcium channel blocker	4 (20)
Nitrates	5 (25)
Ivabradine	4 (20)
Trimetazidine	7 (35)
Diabetic medications, n (%)	
Insulins	5 (25)
Oral hypoglycaemics, n (%)	
Metformin	3 (15)
Sulfonylureas	1 (5)
Glucagon-like peptide-1 receptor agonists	0 (0)
Dipeptidyl peptidase-4 inhibitors	0 (0)
Sodium–glucose cotransporter-2 inhibitors	1 (5)
Cardiovascular procedures, n (%)	
Percutaneous coronary intervention	5 (25)
Coronary artery bypass grafting	3 (15)
PRUs, n (%)	
PRU >208	12 (60)
PRU <208	8 (40)
ARUs, n (%)	
ARU >550	15 (75)
ARU <550	5 (25)
Basic laboratory values	
Serum haemoglobin (g/dL)	13.4 (normal 13.2–17.6)
Serum creatinine (mg/dL)	0.92 (normal 0.81–1.21)
Serum triglycerides (mg/dL)	178 (normal <150)
Serum total cholesterol (mg/dL)	214 (normal <170)
Serum low-density lipoprotein (mg/dL)	162 (normal <130)
Serum high-density lipoprotein (mg/dL)	37 (normal >50)

ARU, aspirin reaction unit; PRU, P2Y12 reaction unit.

**Table 2:** Comparison of patients’ PRUs and ARUs

	Mean PRU	Lower 95% CI	Upper 95% CI	P value
Baseline	215.40	182.31	248.49	0.001
Cocoa Trial	188.55	129.28	192.57	

	Mean ARU	Lower 95% CI	Upper 95% CI	P value
Baseline	485.85	446.58	525.12	0.351
Cocoa Trial	468.20	426.47	509.93	

ARU, aspirin reaction unit; PRU, P2Y12 reaction unit.

## Discussion

Cocoa (*T. cacao* L.) is derived from the cacao bean and has a storied history of diverse therapeutic benefits, including vascular effects based on its flavanol, procyanidin and methylxanthine content.^18–20^ It has substantially contributed to the socioeconomic development of Trinidad and Tobago for over 200 years, at which one point it was responsible for ‘producing 20% of the world’s cocoa’. 21 In fact, as of 1930, the Cocoa Research Centre had pioneered and innovated this sector, making it the oldest cocoa research institution globally.^22^

Cocoa products contain catechins ((+)-catechin) and epicatechins ((−)-epicatechin), classified as flavanols (flavan-3-ols), which display accentuated vascular benefits. They possess a much higher flavanol concentration than wine, tea or berries.^6^ Several nutraceutical studies have been inconsistent in demonstrating a definitive CV benefit, largely attributed to the ambiguity of flavanol concentration.^11^ The recently published ‘Effect of cocoa flavanol supplementation for the prevention of CVD events: the COcoa Supplement and Multivitamin Outcomes Study’ randomised clinical trial revealed no significant effect on the primary outcome of total CV events; however, CV mortality was significantly reduced by 27%.^10^ Numerous mechanistic studies evaluating cocoa have also alluded to enhanced cardiometabolic effects with respect to endothelial function, blood pressure, inflammation, insulin resistance and platelet reactivity.^23–27^

When activated, platelets adhere to sites of vascular injury within a complex milieu of factors promoting aggregation and stabilisation of the haemostatic plug.^28^ These factors include ADP, thromboxane A2 (TXA2), serotonin, collagen (COL) and thrombin.^29^ The release of ADP and TXA2 leads to several mechanistic, morphological and proinflammatory effects, including change in shape, increased expression of P-selectin, soluble CD40 ligand and conversion of the glycoprotein (GP) IIb/IIIa receptor into its active form.^30^

ASA is an irreversible cyclooxygenase-1 (COX-1) inhibitor that blocks TXA2 production. TXA2 is produced from arachidonic acid (AA) through enzymatic conversion by COX-1 and thromboxane synthase. TXA2 binds to the thromboxane receptors, resulting in platelet shape and aggregation of platelets to the primary platelet plug.^31^ By preventing the formation of TXA2, ASA decreases platelet activation and aggregation promoted by TXA2 but not by other agonists.^32^ In this study, there was a non-significant reduction in ARU of 17.65. The VN–ARU assay uses AA as an agonist (sensitive to ASA therapy) and expresses results in ARUs. Despite this study not demonstrating any significant effect of cocoa on ARU with the VN system, Rein *et al* demonstrated that consumption of cocoa caused an ‘ASA-like’ effect on platelet function, as measured in terms of platelet-related primary haemostasis via the platelet function analyser (PFA-100; Siemens Healthineers AG, Erlangen, Germany).^33^ This alternative analyser measures COL–ADP-stimulated or COL–epinephrine (EPI)-stimulated platelet function under shear conditions.^34^ The COL–EPI system detected qualitative platelet abnormalities induced by ASA and was prolonged 6 hours after consuming the cocoa beverage, suggesting an antiplatelet effect.^33^ In that study, the 30 participants consumed 300 mL of a beverage containing 18.75 g cocoa powder, whereas our study included 20 patients consuming 30 g/day of 65% cocoa for 1 week and evaluated with a different platelet function assay.

The consumption of the cocoa intervention significantly decreased PRU by 26.85, with a relative reduction of 11.9%. The VN-P2Y_12_ assay reports results as P2Y_12_ reaction units (PRUs). This assay mimics turbidimetric aggregation and uses disposable cartridges containing 20 mM ADP and 22 nM prostaglandin E1 (PGE1). Aggregation testing using ADP as a sole agonist activates P2Y_1_ and P2Y_12_ purinergic signalling, while adding PGE1 increases the test’s specificity for P2Y_12_ signalling. A baseline value for platelet function is obtained in a separate channel of the cartridge in which isothrombin receptor activating peptide (TRAP) is used as an agonist.^35 36^ The VN system exhibits moderate concordance with other platelet function tests and has reliably stratified high-risk patients for MACE.^37^

Platelet morphology and transient aggregation are mediated by P2Y_1_. Binding of ADP to the P2Y_12_ receptor results in cascade amplification that culminate in platelet aggregation and stabilisation.^38^ In Ostertag *et al*, cocoa significantly decreased ADP-induced platelet aggregation, TRAP-induced platelet aggregation and P-selectin expression, and increased COL/EPI-induced ex vivo bleeding times.^39^ Their methodology included a 60 g cocoa (dark chocolate) bar assessed 6 hours after consumption with platelet function via a PFA-100 analyser and flow cytometry. Additionally, Pearson *et al* demonstrated that cocoa inhibited several measures of platelet activity, including EPI-induced and ADP-induced GP IIb/IIIa and P-selectin expression, platelet microparticle formation, and EPI/COL and ADP-COL induced primary haemostasis.^40^ Montagnana *et al* revealed a significant increase of COL/ADP-induced PFA-100 closure time, but not COL/EPI, 4 hours after ingestion of dark chocolate.^5^ Platelet aggregation induced by COL was unchanged after low flavanol or high flavanol dark chocolate, whereas both attenuated responses to ADP and TRAP relative to baseline.^41^ Shear stress-dependent platelet adhesion was also attenuated in a study by Flammer *et al* using another modality of platelet function testing.^42^ Platelet hyper-reactivity is critical in acute coronary syndrome pathophysiology; thus, mitigation of shear stress-dependent platelet adhesion may beneficially affect atherothrombosis.^42^

Our study displayed a potentiated effect with respect to ADP-induced platelet aggregation in patients on DAPT with ASA and clopidogrel. These comparative studies were performed in patients without established CVD, whereas our study involved patients with a medical history of prior MI, type 2 diabetes mellitus, and who received PCI and CABG.

The P2Y12 receptor and COX-1 pathway are complementary with respect to platelet inhibition, and thus it is surprising that the ARU and PRU signals were not significantly concordant in this study. This could result from an unknown confounder, an interaction effect, or reduced intrinsic power of the study with respect to the number of participants enrolled and the duration of the cocoa intervention. Of the 20 subjects, 5 (25%) had a baseline ARU of >550 which remained unchanged post cocoa intervention, while 12 (60%) had a baseline PRU of >208 compared with 8 (40%) subjects post cocoa intervention, alluding to a non-significant reduction in HPR.

### Study limitations

Despite this study being sufficiently powered for prospective pharmacodynamic outcomes with respect to PRUs and ARUs, it was not designed for prespecified clinical outcomes, and thus no definitive conclusions on clinical efficacy and safety can be ascertained. As with previous studies conducted by this group in Trinidad, there was a preponderance of South Asian patients, alluding to a selection bias during study enrolment.^14 43^ A double-blind, randomised controlled trial would have been the gold standard for ascertaining the antiplatelet effect of this cocoa intervention; however, there are logistical challenges in executing such methodology in our limited resource setting without a dedicated clinical research organisation.^44^

Additionally, this study did not evaluate the composition of the cocoa (dark chocolate) bars with respect to caloric, glucose, protein and lipid content, as any beneficial antiplatelet effect may be potentially offset by paradoxical glycaemic or lipidaemic effects. However, Hamed *et al* reported improved lipid profiles (low-density lipoprotein reduction of 6%, high-density lipoprotein increase of 9%) with decreased platelet reactivity.^45^ Also, this study did not quantify flavanol concentrations with relatively low bioavailability, and their downstream plasma metabolite concentrations are temporally variable and may not correlate with other nutraceutical mechanistic studies.^5^ Our study also involved the cocoa intervention being distributed throughout the day (three 10 g bars consumed at breakfast, lunch and dinner), which may affect the pharmacodynamic effects of the flavanol metabolites on platelet function as compared with the form and timing in other studies, for example, one-time beverage consumption. This cocoa intervention was also relatively short, with a time frame of 1 week, and each cocoa bar contained 65% cocoa solids and 35% sugar. As a result, thus, there can be no long-term extrapolation of clinical and biochemical outcomes such as glycaemic control in patients with diabetes, dyslipidaemic effects or net weight gain, which can have negative implications.

To our knowledge, this prospective study is novel in determining the effect of a 1-week trial of 30 g/day of 65% cocoa consumption intervention on platelet reactivity using the VN system on patients with CAD on DAPT. As such, it may not be clinically pertinent to patients on more potent antithrombotic therapies such as prasugrel, ticagrelor or direct oral anticoagulants. Additionally, many of these therapies and their generic counterparts are not readily available due to regulatory or financial issues. A more inclusive and detailed array of platelet function testing using PFA, flow cytometry and thromboelastography may be revelatory; however, these are unavailable in Trinidad due to technical and personnel logistical issues.

## Conclusions

Significantly attenuated platelet reactivity was observed with the cocoa intervention with respect to PRUs but not with ASA reaction units. This nutraceutical study could be potentially informative and applicable for patients with stable CAD. Further long-term studies are required to confirm these exploratory findings.

## Data Availability

Data are available upon reasonable request.
